# Elevated serum fibrinogen level is an independent risk factor for IgA nephropathy

**DOI:** 10.18632/oncotarget.21702

**Published:** 2017-10-09

**Authors:** Ji Zhang, Chaosheng Chen, Qiongxiu Zhou, Shubei Zheng, Yinqiu Lv, Jianna Zhang, Xiaohan You, Zhanyuan Li, Zhihong Zhou, Min Pan

**Affiliations:** ^1^ Department of Nephrology, The Second Affiliated Hospital and Yuying Children's Hospital of Wenzhou Medical University, Wenzhou, Zhejiang, P.R. China; ^2^ Department of Nephrology, The First Affiliated Hospital of Wenzhou Medical University, Wenzhou, Zhejiang, P.R. China

**Keywords:** IgA nephropathy, fibrinogen, chronic kidney disease, Oxford classification, renal outcome

## Abstract

**Background:**

IgA nephropathy is a primary cause of renal failure, and inflammation and renal fibrosis are the main mechanisms leading to kidney damage. The serum fibrinogen level is closely related to inflammatory states, but its relationship to the prognosis of IgA nephropathy (IgAN) is unclear.

**Materials and Methods:**

1053 patients diagnosed with IgAN after renal biopsy were enrolled from two Nephrology Departments. Demographic and clinical data and histopathological features were collected. The patients were divided into four groups (Q1–Q4) according to the serum fibrinogen levels at the time of renal biopsy, and the relationships of serum fibrinogen levels with other risk factors and the prognosis of IgAN were investigated.

**Results:**

672 patients with proven primary IgAN were included in this study, which included a median follow-up of 36 months. Patients with higher serum fibrinogen levels had elevated serum creatinine levels, 24-hour urinary protein, and blood pressure compared with patients with the lowest levels of serum fibrinogen as well as severe renal damage at the time of renal biopsy. Univariate and multivariate Cox regression analyses confirmed that the serum fibrinogen level at the time of renal biopsy was significantly related to the prognosis of patients with IgAN.

**Conclusions:**

In patients with IgAN, an elevated serum fibrinogen level at the time of renal biopsy is associated with poor renal outcomes, which suggests the need for more aggressive early interventions. Greater benefits of aggressive treatments were observed in patients with higher serum fibrinogen levels.

## INTRODUCTION

IgA nephropathy (IgAN) is the most common type of primary glomerulonephritis worldwide and is characterized by the deposition of IgA in the glomerular mesangium. Approximately one-third of patients with IgAN develop end-stage renal disease within 20–30 years after their initial diagnosis [1].

Nephrologists find it challenging to predict the prognosis of and identify appropriate treatment options for patients with IgAN. The clinical courses of patients with IgAN are highly variable, and the early identification of patients who will likely exhibit poor renal outcomes and should thus receive aggressive treatment remains difficult. The Oxford classification of IgAN based on renal histopathology is considered the strongest early predictive histological risk factor for patients with IgAN [2], and the urinary protein and serum creatinine levels at the time of renal biopsy are also strong clinical predictive factors [3, 4]. However, IgAN is considered an autoimmune disease and is accompanied by inflammatory activity [5]. These traditional indicators do not directly reflect the patient's inflammatory status, and the roles of inflammatory factors in the prognosis and determination of treatment options are unclear.

Accumulating evidence supports the notion that fibrinogen, a plasma glycoprotein that is elevated in patients with acute inflammatory conditions, plays a critical role in regulating the inflammatory process, and increased serum fibrinogen levels are considered both an indicator of a proinflammatory status and a high-risk marker for the development of vascular inflammatory diseases [6]. The serum fibrinogen levels are also related to increased blood viscosity, which causes endothelial shear-stress damage [7]. However, to the best of our knowledge, the association between the serum fibrinogen level and the prognosis of patients with IgAN has not been investigated. The aim of this study was to evaluate the role of serum fibrinogen levels in the early prediction of poor renal outcomes and to determine whether the serum fibrinogen levels can be used to identify treatment options for patients with IgAN.

## RESULTS

### Participants

**Figure 1:**
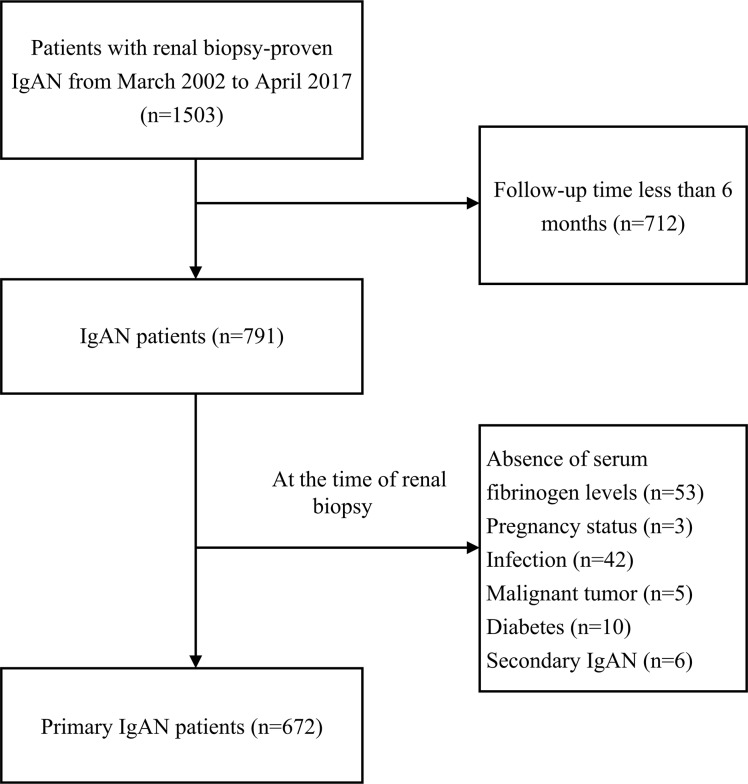
Flow chart of the strategy used to identify patients with IgAN

One thousand fifty-three patients diagnosed with IgAN by renal biopsy were initially enrolled in the study, and 672 of these patients met the inclusion criteria (Figure 1). The median age of the included patients was 36 years (range 28–45 years), and 383 (57%) were female. The median follow-up time was 36 months (range 19–61 months). The primary renal outcome was observed in 13.4% (*n* = 90) of the patients, and 4.5% (*n* = 30) of the patients developed end-stage renal failure (ESRD) at the end of the follow-up period.

### Relationships between serum fibrinogen levels and clinical or histopathological features

The clinical characteristics and histopathological features of 672 patients separated by quartiles of serum fibrinogen levels at the time of renal biopsy and during follow-up are shown in Table 1. No significant differences in the sex distribution, serum high-density lipoprotein cholesterol (HDL-C) levels, and follow-up time were observed among these four groups. Patients with higher serum fibrinogen levels were older, had higher body mass indexes (BMIs), and displayed significantly increased total cholesterol (TC), triglyceride (TG), low-density lipoprotein cholesterol (LDL-C), serum creatinine (Scr), and uric acid (UA), 24-hour urinary protein (Upro), and systolic and diastolic blood pressure (SBP and DBP, respectively) levels and significantly decreased serum albumin (ALB) and hemoglobin (Hb) levels. According to the MEST-C scoring system, in patients with M1 and T1/T2 scores had higher serum fibrinogen levels, but no differences were observed among patients with E, S, and C scores. During follow-up, no clear differences in the percentages of patients receiving ACEI or ARB treatments were observed among the quartiles. However, a higher proportion of patients with higher fibrinogen levels received immunosuppressive therapies, and these patients were more likely to develop poor renal outcomes at the end of the follow-up period. The incidence of primary endpoint conditions and ESRD gradually increased from Q1 to Q4 (1.8%, 5.4%, 17.4%, and 29.7% and 1.2%, 0.6%, 6.0%, and 10.3%, respectively; both *p*-values < 0.001).

**Table 1:** Clinical characteristics and histopathological features of the patients enrolled in this study stratified according to quartiles of serum fibrinogen levels

Characteristics	Serum fibrinogen levels, g/l				*p*-value
	Q1 (1.7–2.8)	Q2 (2.8–3.3)	Q3 (3.3–3.9)	Q4 (3.9–8.2)	
Patients (n)	171	167	167	165	
**At time of biopsy**					
Female (n [%])	95 (55.6)	100 (59.9)	99 (59.3)	87 (52.7)	0.515
Age (mean [SD], years)	32.3 (8.3)	36.4 (11.2)	38.4 (11.4)	41.9 (13.9)	< 0.001
BMI (mean [SD], kg/m^2^)	22.0 (2.9)	23.00 (3.2)	23.12 (3.3)	24.0 (3.8)	< 0.001
TC (mean [SD], g/l)	4.4 (0.9)	4.9 (1.1)	5.1 (1.1)	5.7 (1.7)	< 0.001
TG (median [IQR], g/l)	1.2 [0.9, 1.6]	1.5 [1.1, 2.3]	1.6 [1.1, 2.2]	1.9 [1.3, 2.5]	< 0.001
HDL-C (mean [SD], g/l)	1.2 (0.3)	1.2(0.3)	1.1 (0.3)	1.1 (0.3)	0.521
LDL-C (mean [SD], g/l)	2.6 (0.7)	2.9 (0.9)	3.0 (0.8)	3.4 (1.3)	< 0.001
Scr (median [IQR], mmol/l)	73 [59, 90]	73 [59, 96]	83 [63, 113]	98 [75, 138]	< 0.001
Upro (median [IQR], g/24 h)	0.8 [0.4, 1.2]	1.0 [0.6, 1.6]	1.4 [0.9, 2.9]	2.7 [1.5, 4.8]	< 0.001
eGFR (mean [SD], ml/min/1.73 m^2^)	105 (25.2)	99 (25.5)	86 (30.8)	71 (31.9)	< 0.001
ALB (mean [SD], g/l)	40.3 (6.7)	40.0 (6.7)	38.1 (7.3)	34.2 (8.3)	< 0.001
UA (median [IQR], mmol/l)	342 [274, 404]	355 [289, 414]	355 [305, 440]	382 [327, 446]	< 0.001
Hb (mean [SD], g/l)	125.1 (19.3)	130.3 (17.0)	125.5 (18.5)	121.6 (19.5)	< 0.001
SBP (mean [SD], mmHg)	124.96 (15.8)	130.41 (21.3)	132.29 (16.9)	137.98 (19.8)	< 0.001
DBP (mean [SD], mmHg)	77.25 (10.3)	81.34 (12.1)	81.58 (12.4)	84.61 (12.0)	< 0.001
**Oxford classification**					
M1 (n [%])	60 (35.9)	72 (43.4)	75 (45.7)	94 (57.7)	0.001
E1 (n [%])	55 (32.9)	38 (22.9)	51 (30.9)	48 (29.4)	0.206
S1 (n [%])	129 (77.2)	124 (75.2)	134 (81.2)	119 (73.0)	0.338
T (n [%])					< 0.001
T0	130 (77.8)	119 (71.3)	89 (53.9)	81 (49.7)	
T1	30 (18.0)	38 (22.8)	54 (32.7)	39 (23.9)	
T2	7 (4.2)	10 (6.0)	22 (13.3)	43 (26.4)	
C1 (n [%])	79 (46.5)	65 (38.9)	82 (49.1)	86 (52.8)	0.077
**Follow-up**					
Follow-up time (median [IQR], months)	41.0 [20.0, 66.5]	34.0 [19.5, 55.5]	34.0 [16.0, 57.5]	35.0 [20.0, 64.0]	0.034
Primary endpoint (n [%])	3 (1.8)	9 (5.4)	29 (17.4)	49 (29.7)	< 0.001
ESRD (n [%])	2 (1.2)	1 (0.6)	10 (6.0)	17 (10.3)	< 0.001
Treated with ACEIs (n [%])	65 (38.0)	54 (32.3)	66 (39.5)	57 (34.5)	0.509
Treated with ARBs (n [%])	148 (86.5)	148 (88.6)	147 (88.0)	133 (80.6)	0.136
**Immunosuppressant therapy**					
Glucocorticoids (n [%])	65 (38.0)	79 (47.3)	95 (56.9)	98 (59.4)	< 0.001
Cyclophosphamide (n [%])	6 (3.5)	3 (1.8)	14 (8.4)	25 (15.2)	< 0.001
OtherIM (n [%])	36 (21.1)	44 (26.3)	48 (28.7)	61 (37.0)	0.012

The difference between the clinical and histopathological parameters separated by the quartiles of serum fibrinogen levels were calculated using the Kruskal–Wallis test for numerical variables and Pearson's Chi-squared test for categorical variables.

### Risk factors for poor renal outcomes

The univariate Cox proportional hazard regression analysis (Model 1) results are shown in Table 2. Patients who were older, male, and had increased levels of TC, TG, LDL-C, Scr, Upro, UA, SBP, and DBP and decreased levels of HDL-C, ALB and Hb were at greater risk of poor renal outcomes. In reference to Q1, the HRs for poor renal outcomes in the Q2, Q3, and Q4 patients were 4.15 (95% CI 1.12–15.34, *p*-value = 0.033), 11.43 (95% CI 3.48–37.56, *p*-value < 0.001), and 17.15 (95% CI 5.34–55.07, *p*-value < 0.001). Analysis of the MEST-C scoring system identified M1 (in reference to M0, HR 2.3, 95% CI 1.47–3.6, *p*-value < 0.001), T1 (in reference to T0, HR 3.24, 95% CI 1.91–5.48, *p*-value < 0.001), and T2 (in reference to T0, HR 10.43, 95% CI 6.09–17.84, *p*-value < 0.001) as risk factors for poor outcomes. Interestingly, in our cohort, C1 (in reference to C0, HR 0.62, 95% CI 0.4–0.97, *p*-value = 0.038) was a protective factor for renal outcomes.

**Table 2:** Univariate and multivariate Cox proportional hazard analyses of the risk of a 50% decrease in the eGFR or ESRD

Characteristics	Model 1			Model 2			Model 3		
	HR	95% CI	*p*-value	HR	95% CI	*p*-value	HR	95% CI	*p*-value
**Gender**									
Male	1.00	(Reference)		1.00	(Reference)		1.00	(Reference)	
Female	0.45	0.29–0.69	< 0.001	1.00	0.51–1.95	0.994	1.27	0.61–2.65	0.526
Age	1.02	1.01–1.04	0.012	0.99	0.97–1.02	0.659	1.00	0.97–1.02	0.833
BMI	0.99	0.93–1.06	0.842						
TC	1.20	1.06–1.37	0.005	0.80	0.55–1.17	0.255	0.83	0.53–1.3	0.425
TG	1.22	1.06–1.4	0.005	1.14	0.88–1.46	0.325	1.03	0.77–1.38	0.847
HDL-C	0.33	0.15–0.7	0.004	0.74	0.26–2.11	0.569	0.71	0.22–2.28	0.569
LDL-C	1.28	1.05–1.56	0.013	0.99	0.65–1.52	0.978	0.99	0.61–1.61	0.969
Scr	1.01	1.01–1.01	< 0.001	1.01	1–1.01	0.006	1.00	1–1.01	0.079
Upro	1.28	1.2–1.37	< 0.001	1.05	0.91–1.21	0.529	1.05	0.9–1.22	0.566
ALB	0.91	0.89–0.94	< 0.001	0.92	0.87–0.97	0.003	0.93	0.88–0.98	0.008
UA	1.01	1.01–1.01	< 0.001	1.00	1–1.01	0.012	1.00	1–1.01	0.011
Hb	0.99	0.98 – 0.99	0.024	1.01	0.99–1.02	0.614	1.01	0.99–1.04	0.179
SBP	1.02	1.01–1.03	< 0.001	1.01	0.99–1.03	0.233	1.01	1–1.03	0.121
DBP	1.04	1.02–1.05	< 0.001	1.01	0.98–1.04	0.429	1.01	0.98–1.04	0.723
**Quartile of fibrinogen levels**									
Q1	1.00	(Reference)		1.00	(Reference)		1.00	(Reference)	
Q2	4.15	1.12–15.34	0.033	2.62	0.66–10.41	0.170	2.10	0.52–8.43	0.294
Q3	11.43	3.48–37.56	< 0.001	4.78	1.36–16.86	0.015	3.66	1.02–13.16	0.047
Q4	17.15	5.34–55.07	< 0.001	5.50	1.55–19.54	0.008	4.78	1.31–17.39	0.018
**Oxford classification**									
M0	1.00	(Reference)					1.00	(Reference)	
M1	2.30	1.47–3.6	< 0.001				1.50	0.87–2.57	0.146
E0	1.00	(Reference)							
E1	0.76	0.45–1.28	0.305						
S0	1.00	(Reference)							
S1	1.57	0.89–2.79	0.121						
T0	1.00	(Reference)					1.00	(Reference)	
T1	3.24	1.91–5.48	< 0.001				1.81	0.96–3.4	0.065
T2	10.43	6.09–17.84	< 0.001				2.84	1.39–5.8	0.004
C0	1.00	(Reference)					1.00	(Reference)	
C1	0.62	0.4–0.97	0.038				0.82	0.47–1.42	0.480

Model 1 is a univariate analysis model. Model 2 is adjusted for the following clinical parameters: age, TC, TG, LDL-C, Scr, Upro, ALB, UA, Hb, BMI, SBP, and DBP. Model 3 is adjusted for the above-mentioned clinical parameters and MEST-C scores.

**Figure 2:**
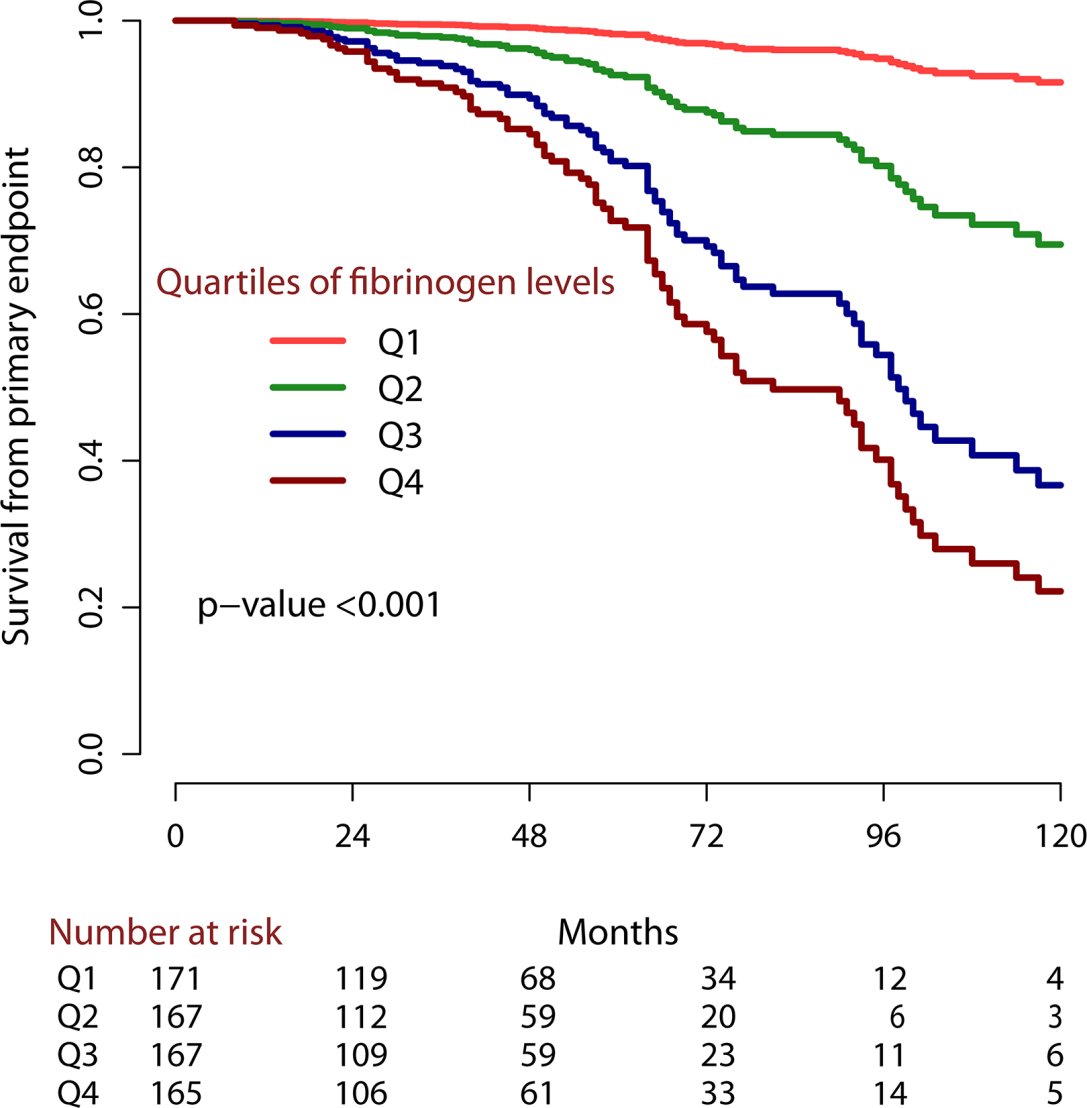
Kaplan-Meier survival analysis according to quartiles of serum fibrinogen levels The plot was prepared using the *rms package*. The numbers of patients at risk of poor renal outcomes are shown under the plot.

A Kaplan-Meier survival analysis was performed to examine the correlation between the four quartiles of fibrinogen levels and renal survival in patients with IgAN (Figure 2). The estimated 5-year and 10-year survival rates for patients with renal outcomes gradually decreased from Q1 to Q4 (0.99 and 0.93, 0.92 and 0.81, 0.8 and 0.36, 0.74 and 0.22, respectively).

Two multivariate Cox proportional hazard regression models were calculated for variables with *p*-values ≤ 0.10 in the univariate analysis. According to model 2, which included the clinical parameters, Scr, ALB, UA, Q3 and Q4 were independent risk factors for renal survival. Model 3, which included the clinical parameters and MEST-C scores, showed that ALB, UA, Q3, Q4 and T2 were independent risk factors for endpoint conditions (Table 2).

### Role of treatment in the prognosis of patients with IgAN

During follow-up, approximately 50% of the patients received glucocorticoids (GS), 7.1% received cyclophosphamide (CTX), and 28.1% received other immunosuppressive agents (OtherIM), including leflunomide, mycophenolate mofetil, and calcineurin inhibitors. A higher proportion of patients in the group with higher fibrinogen levels received immunosuppressive therapy (*p*-values: < 0.001 for GS, < 0.001 for CTX, and 0.012 for OtherIM). Approximately 36% and 85.7% of the patients received angiotensin-converting enzyme inhibitors (ACEIs) and angiotensin receptor blockers (ARBs), respectively, but the differences in the proportions among groups with different fibrinogen levels were not statistically significant (*p*-values: 0.509 and 0.136, respectively).

**Figure 3:**
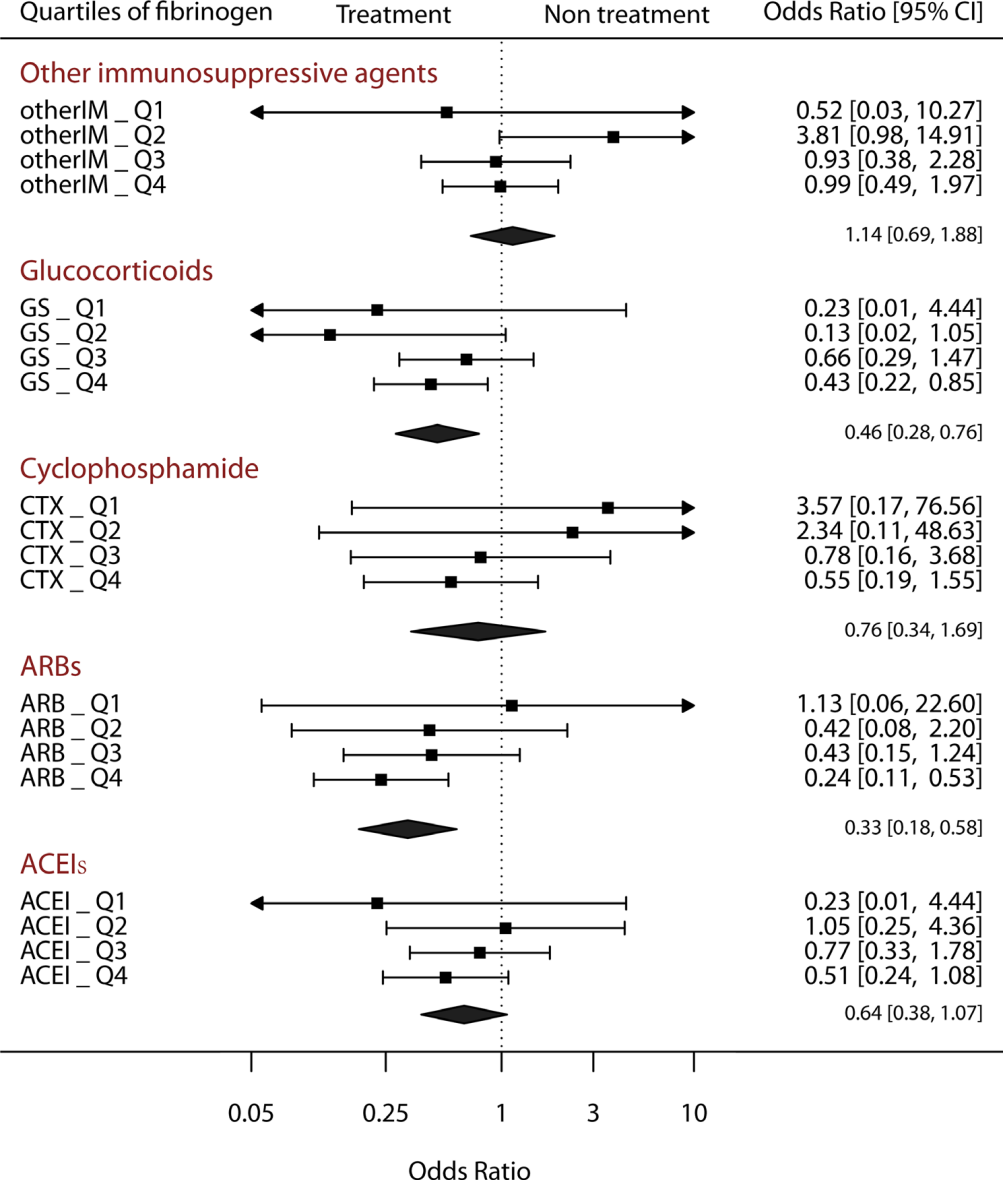
Forest plot of the relationship between treatments and quartiles of serum fibrinogen levels

A forest plot was prepared to assess the role of treatment in the prognosis of IgAN patients with fibrinogen levels in the different quartiles (Figure 3). In the immunosuppressive therapy group, GS therapy significantly improved the prognosis of patients with IgAN (Combined OR 0.46, 95% CI 0.28–0.76, *p*-value 0.002), and a higher effect was observed in patients with higher fibrinogen levels. Although the combined effect of CTX therapy was not significantly different (combined OR 0.76, 95% CI 0.34–1.69, *p*-value 0.494), different effects were observed between patients with low (OR 3.57 and 2.34 and 95% CI 0.17–76.56 and 0.11–48.63 for the Q1 and Q2 groups, respectively) and high fibrinogen levels (OR 0.78 and 0.55 and 95% CI 0.16–3.68 and 0.19–1.55 for the Q3 and Q4 groups, respectively). In the OtherIM group, the treatment did not appear to improve the prognosis of patients with IgAN (combined OR 1.14, 95% CI 0.69–1.88, *p*-value 0.603). Moreover, treatment with ACEIs (combined OR 0.64, 95% CI 0.38–1.07, *p*-value 0.087) and ARBs (combined OR 0.24, 95% CI 0.18–0.58, *p*-value < 0.001) improved the prognosis of the enrolled cohort.

### Sensitivity analyses

The enrolled cohort exhibited a propensity toward treatment with immunosuppressive agents across the different quartiles of serum fibrinogen levels (Table 1). Therefore, a propensity score matching (PSM) analysis method was performed in an attempt to reduce the bias associated with the use of immunosuppressive therapies, such as glucocorticoids, cyclophosphamide, and other immunosuppressive agents, as well as the follow-up duration, with the aim of examining the relationships between the four quartiles of fibrinogen levels and poor renal outcomes. Our univariate and multivariate logistic regression models were adjusted for clinical variables and MEST-C scores, and the findings showed that an increased serum fibrinogen level was associated with a significant increase in the incidence of primary endpoint conditions (Table 3).

**Table 3:** Propensity score matching analysis to evaluate the relationships between quartiles of serum fibrinogen levels and poor renal outcomes

Quartiles of fibrinogen	Model 1			Model 2			Model 3		
	OR	95% CI	*p*-value	OR	95% CI	*p*-value	OR	95% CI	*p*-value
Q1	1.0	(Reference)		1.0	(Reference)		1.0	(Reference)	
Q2	3.5	0.98–16.6	0.071	2.9	0.6–16.5	0.187	2.6	0.5–15.3	0.258
Q3	15.0	4.8–66.8	< 0.001	9.6	2.4–51.7	0.003	8.9	2.1–49.9	0.005
Q4	27.0	8.8–118.5	< 0.001	14.0	3.2–80.2	0.001	13.0	3.0–80.3	0.001

Model 1 is a univariate analysis model. Model 2 is adjusted for the following clinical parameters: age, TC, TG, LDL-C, Scr, Upro, ALB, UA, Hb, BMI, SBP, and DBP. Model 3 is adjusted for the above-mentioned clinical parameters and MEST-C scores.

## DISCUSSION

The serum fibrinogen level has recently been confirmed to be associated with poor outcomes in patients with CKD [8, 9]. However, to the best of our knowledge, our study constitutes the first multicenter, real-world study that enrolled a large cohort and performed long-term follow-up to assess the role of the serum fibrinogen level at the time of renal biopsy in early prognosis prediction and to determine whether the serum fibrinogen level can be used to identify treatment options for patients with IgAN.

Fibrinogen is a large hexameric plasma glycoprotein synthesized in the liver, and its levels are significantly increased in patients with acute inflammatory conditions. Fibrinogen is considered a key regulator of inflammation in disease [10]. Previous studies have confirmed that fibrinogen binds to different integrin receptors expressed in leukocytes, macrophages, mononuclear cells, platelets, and other inflammatory cells, and fibrinogen activates cell signaling pathways, such as the nuclear factor κB (NF-κB) and mitogen-activated protein kinase (MAPK) signaling pathways, to promote transforming growth factor β (TGF-β) synthesis and activation and subsequently enhance inflammatory processes [11, 12]. These inflammatory pathways and inflammatory factors have also been confirmed to be key mediators of renal inflammation and fibrosis [13, 14]. Fibrinogen directly stimulates normal rat kidney interstitial fibroblast proliferation *in vitro* in a dose-dependent manner, and fibrinogen deficiency significantly protects a mouse model of unilateral urethral obstruction from interstitial damage and tubular disruption, attenuates collagen accumulation, and substantially reduces *de novo* expression of α-smooth muscle actin in the obstructed kidney [15]. In a mouse model heterozygous for modified fibrinogen (< 75% of the normal plasma fibrinogen levels), only 3% of animals exhibited kidney interstitial fibrosis and tubular atrophy compared with 24% of wild-type mice [14]. As a damage-associated mitogenic signal, fibrinogen also directly damages podocytes, leading to glomerular sclerosis and promoting glomerular mesangial cell proliferation and tubulointerstitial fibrosis [15–20].

The Chronic Renal Insufficiency Cohort (CRIC) Study investigated a variety of inflammatory factors in plasma, such as interleukin-1 (IL-1), IL-1 receptor antagonist, IL-6, tumor necrosis factor-α, transforming growth factor-β, high-sensitivity C-reactive protein, and fibrinogen and showed that elevated serum fibrinogen levels are associated with a rapid loss of kidney function in patients with CKD [21]. According to other clinical studies, higher serum fibrinogen levels are associated with a lower estimated glomerular filtration rate (eGFR) in patients with CKD [22–24]. In our cohort, we found a significant correlation between the serum fibrinogen and CRP levels (Pearson's correlation coefficient = 0.44, *p*-value < 0.001) at the time of renal biopsy.

In our study, the incidences of a 50% decrease in the eGFR and the development of ESRD were significantly higher, and the 5- and 10-year kidney survival rates were significantly lower in patients with IgAN who displayed higher serum fibrinogen levels than in patients with lower serum fibrinogen levels. It is acknowledged that the presence of cellular crescents is an acute pathological indicator of renal damage [25]. However, only those patients who did not receive immunosuppressive therapy showed a statistically significant association between crescents and poor renal outcomes [26, 27]. In our cohort, a higher proportion of patients with a C1 score received treatment with glucocorticoids and CTX compared with patients with a C0 score (59.7% vs. 41.6% and 9.3% vs. 5.3%, *p*-values < 0.001 and 0.67, respectively). Therefore, the protective effect of the C score might be due to the fact that patients with a C1 score are more likely to receive aggressive immunosuppressive therapies. It has been suggested that patients with obvious inflammatory activity should be administered aggressive immunosuppressive therapy. Multivariate Cox proportional hazard models adjusted for clinical factors and histopathological risk factors according to the Oxford classification of IgAN also confirmed that elevated fibrinogen levels at the time of renal biopsy are an independent risk factor for poor prognoses of patients with IgAN, and the predictive ability of this factor might be stronger than those of the serum creatinine levels and urinary protein excretion, which are considered clinical risk factors that strongly predict IgAN. A propensity score matching analysis was performed to reduce the bias associated with treatments and follow-up time, and the results further confirmed that higher serum fibrinogen levels are associated with a higher incidence of poor kidney outcomes.

The current treatment for IgAN primarily includes antiproteinuric and antihypertensive therapies using ACEIs or ARBs and immunosuppressive therapy using glucocorticoids, cyclophosphamide, and other immunosuppressive agents, such as mycophenolate mofetil, acetazolamide, and calcineurin inhibitors [8, 9]. Although the Kidney Disease: Improving Global Outcomes (KDIGO) guidelines provide a detailed treatment protocol, these guidelines have low recommendation levels and weak evidentiary quality; moreover, the rationale for the treatment guidelines is primarily based on the urinary protein levels.

Researchers have not clearly determined whether the serum fibrinogen levels serve as an indicator of treatment options. Our forest plot showed an obvious relationship between the serum fibrinogen level and treatment strategy. ACEIs and ARBs provide benefits in addition to lowering blood pressure, reducing inflammation status and reducing the release of inflammatory factors such as TGF-β [28–30]. In our ARB- or ACEI-treated cohort, patients with higher fibrinogen levels experienced a greater benefit from treatment. Immunosuppressive therapy is a key treatment for inflammation. Because these drugs might have serious side effects, they are used only under certain conditions. Based on our data, not all immunosuppressive treatments improve the prognosis of patients with IgAN. The combined effect of glucocorticoid therapy significantly reduced the incidence of poor renal outcomes, particularly in the group of patients with high fibrinogen levels. In the cyclophosphamide-treated group, the treatment exerted a protective effect on patients with higher serum fibrinogen levels, whereas the treatment might have increased the incidence of poor renal outcomes in patients with lower serum fibrinogen levels. No clear therapeutic effects were observed in the group administered other immunosuppressive agents. Therefore, our results indicate that patients with higher serum fibrinogen levels should receive treatment with ARBs, ACEIs, and glucocorticoids. These results are consistent with the results from other studies based on the urinary protein levels [4, 31].

Our study has certain limitations. First, many patients were lost during follow up, and our study lacked unified treatment regimens. Although we performed propensity score matching to correct for the bias associated with treatment and follow-up time, bias might still affect the robustness of our results. Second, because the serum fibrinogen level is a nonspecific inflammatory marker, it might be affected by other factors, such as acute infection, malignant neoplasms, and other complicating diseases, such as diabetes. We initially excluded patients with these diseases from our study, but other factors might still confound our results. Third, serum fibrinogen levels were not measured during the follow-up period because their measurement is not routinely performed; however, a single baseline test has been reported to accurately reflect an individual's inflammatory status over time [32].

To summarize, in patients with IgAN, an elevated serum fibrinogen level at the time of renal biopsy is associated with poor renal outcomes and suggests the need for early aggressive intervention. Therapy with ACEIs, ARBs, and glucocorticoids improve patient prognosis, and patients with higher serum fibrinogen levels experience greater benefits from these treatments. However, additional, more rigorous studies are needed to confirm these results.

## MATERIALS AND METHODS

### Subjects

Patients’ clinical data were collected from hospital databases if they received a diagnosis of biopsy-proven primary IgAN between 2002 and 2017 and attended follow-up within at least 6 months at The First and Second Affiliated Hospitals of Wenzhou Medical University. Patients who did not receive any corticosteroids or immunosuppressants before renal biopsy were included. The following patients were excluded from the study: 1. patients with secondary IgAN, such as systemic lupus erythematosus, Henoch-Schönlein purpura, or hepatic disease; 2. patients with a history of pregnancy in the three months before renal biopsy; 3. patients with a history of infection in the two weeks before renal biopsy; 4. patients who were complicated with other chronic diseases, such as malignant tumors or diabetic nephropathy; and 5. patients with missing data at the time of renal biopsy.

### Collection of clinical variables

The patients’ data (age and gender) and clinical features, including SBP, DBP, BMI, ALB, Scr, TG, TC, LDL-C, HDL-C, serum fibrinogen levels, UA, Hb and Upro levels, were collected at the time of renal biopsy. The eGFR was calculated using the Chronic Kidney Disease (CKD) Epidemiology Collaboration equation [33]. The use of medications, including immunosuppressants, such as GS, CTX and OtherIM, and antihypertensive and antiproteinuric drugs, such as ACEIs and ARBs, was also recorded. All cases were scored according to the new Oxford classification of the IgAN scoring system (MEST-C scoring system) and defined as follows: M0 and M1 were defined as ≤ and > 50% of glomeruli with mesangial cell proliferation, respectively; E0 and E1 were defined as the presence and absence of endocapillary hypercellularity, respectively; S0 and S1 were defined as the presence and absence of segmental sclerosis or tuft adhesions, respectively; T0, T1, and T2 were defined based on the degree of tubular atrophy or interstitial fibrosis (< 25%, 25–50%, and > 50%, respectively), and C0 and C1 were defined as the presence and absence of cellular/fibrocellular crescents in the glomeruli, respectively [26, 34].

### Statistical analysis

The numerical variables are presented as the means (standard deviations [SD]) or medians with interquartile ranges (IQR), and the categorical variables are presented as counts with percentages (%). The baseline demographic and clinical characteristics of the included patients and the histopathological data were separated into four groups according to the serum fibrinogen level quartiles and were compared using the Kruskal–Wallis test for numerical variables and Pearson's Chi-squared test for categorical variables. The primary endpoints were poor renal outcomes, defined as a decrease of at most 50% in the eGFR from the baseline level or progression to end-stage renal disease during follow-up. Renal survival was defined as the absence of the primary endpoints during follow-up. Univariate and multivariate odds ratios (ORs) and hazard ratios (HRs) with 95% confidence intervals (CIs) were calculated using a logistic regression model and a Cox proportional hazards model. A propensity score matching analysis was performed using the *MatchIt* package in R [35] to reduce the bias associated with the different treatments and follow-up times, with a 1:2 ratio. All reported *p*-values were two-tailed, and *p*-values less than 0.05 were considered to indicate statistical significance. The analyses were performed using R [36] and R packages [37].

### Ethical approval and consent to participate

This study was performed with the written informed consent of all patients, and the procedures were approved by the Ethics Committee of The Second Affiliated Hospital & Yuying Children's Hospital of Wenzhou Medical University.

## Abbreviations

ALB: serum albumin
SBP: systolic blood pressure
DBP: diastolic blood pressure
ACEIs: angiotensin-converting enzyme inhibitors
ARBs: angiotensin receptor blockers
CIs: 95% confidence Intervals
CKD: Chronic kidney disease
CTX: cyclophosphamide
eGFR: estimated glomerular filtration rate
GS: glucocorticoids
Hb: hemoglobin
HDL-C: high-density lipoprotein cholesterol
HRs: hazard ratios
IQR: interquartile ranges
KDIGO: Kidney Disease Improving Global Outcomes
LDL-C: low-density lipoprotein cholesterol
MAPK: mitogen-activated protein kinase
MEST-C scoring system: the new Oxford classification of the IgAN scoring system
NF-κB: nuclear factor κB
ORs: odds ratios
OtherIM: other immunosuppressive gents
Scr: serum creatinine
SD: standard deviations
TC: total cholesterol
TG: triglyceride
TGF-β: transforming growth factor
UA: uric acid
Upro: 24-hour urinary protein.

## References

[1] Lai KN, Tang SC, Schena FP, Novak J, Tomino Y, Fogo AB, Glassock RJ (2016). IgA nephropathy. Nat Rev Dis Primers.

[2] Barbour SJ, Espino-Hernandez G, Reich HN, Coppo R, Roberts IS, Feehally J, Herzenberg AM, Cattran DC, Oxford Derivation, North American Validation, VALIGA Consortia (2016). The MEST score provides earlier risk prediction in IgA nephropathy. Kidney Int.

[3] Donadio JV, Grande JP (2002). IgA nephropathy. N Engl J Med.

[4] KDIGO Group (2012). KDIGO clinical practice guideline for glomerulonephritis. Kidney Int Supp.

[5] Suzuki H, Fan R, Zhang Z, Brown R, Hall S, Julian BA, Chatham WW, Suzuki Y, Wyatt RJ, Moldoveanu Z, Lee JY, Robinson J, Tomana M (2009). Aberrantly glycosylated IgA1 in IgA nephropathy patients is recognized by IgG antibodies with restricted heterogeneity. J Clin Invest.

[6] Adams RA, Schachtrup C, Davalos D, Tsigelny I, Akassoglou K (2007). Fibrinogen signal transduction as a mediator and therapeutic target in inflammation: lessons from multiple sclerosis. Curr Med Chem.

[7] Lominadze D, Dean WL, Tyagi SC, Roberts AM (2010). Mechanisms of fibrinogen-induced microvascular dysfunction during cardiovascular disease. Acta Physiol (Oxf).

[8] Weiner DE, Tighiouart H, Elsayed EF, Griffith JL, Salem DN, Levey AS, Sarnak MJ (2008). The relationship between nontraditional risk factors and outcomes in individuals with stage 3 to 4 CKD. Am J Kidney Dis.

[9] Mulay SR, Kumar SV, Lech M, Desai J, Anders HJ (2016). How kidney cell death induces renal necroinflammation. Seminars in Nephrology: Elsevier.

[10] Davalos D, Akassoglou K (2012). Fibrinogen as a key regulator of inflammation in disease. Seminars in immunopathology. Springer.

[11] Schachtrup C, Ryu JK, Helmrick MJ, Vagena E, Galanakis DK, Degen JL, Margolis RU, Akassoglou K (2010). Fibrinogen triggers astrocyte scar formation by promoting the availability of active TGF-beta after vascular damage. J Neurosci.

[12] Vidal B, Serrano AL, Tjwa M, Suelves M, Ardite E, De Mori R, Baeza-Raja B, Martinez de Lagran M, Lafuste P, Ruiz-Bonilla V, Jardi M, Gherardi R, Christov C (2008). Fibrinogen drives dystrophic muscle fibrosis via a TGFbeta/alternative macrophage activation pathway. Genes Dev.

[13] Meng XM, Zhang Y, Huang XR, Ren GL, Li J, Lan HY (2015). Treatment of renal fibrosis by rebalancing TGF-beta/Smad signaling with the combination of asiatic acid and naringenin. Oncotarget.

[14] Craciun FL, Ajay AK, Hoffmann D, Saikumar J, Fabian SL, Bijol V, Humphreys BD, Vaidya VS (2014). Pharmacological and genetic depletion of fibrinogen protects from kidney fibrosis. Am J Physiol Renal Physiol.

[15] Sorensen I, Susnik N, Inhester T, Degen JL, Melk A, Haller H, Schmitt R (2011). Fibrinogen, acting as a mitogen for tubulointerstitial fibroblasts, promotes renal fibrosis. Kidney Int.

[16] Wang Y, Zheng C, Xu F, Liu Z (2016). Urinary fibrinogen and renal tubulointerstitial fibrinogen deposition: Discriminating between primary FSGS and minimal change disease. Biochem Biophys Res Commun.

[17] Motojima M, Matsusaka T, Kon V, Ichikawa I (2010). Fibrinogen that appears in Bowman's space of proteinuric kidneys in vivo activates podocyte Toll-like receptors 2 and 4 in vitro. Nephron Exp Nephrol.

[18] Banas MC, Banas B, Hudkins KL, Wietecha TA, Iyoda M, Bock E, Hauser P, Pippin JW, Shankland SJ, Smith KD, Stoelcker B, Liu G, Grone HJ (2008). TLR4 links podocytes with the innate immune system to mediate glomerular injury. J Am Soc Nephrol.

[19] Fischer EG (2004). Glomerular mesangial cell adhesion to fibrinogen is mediated by alphavbeta3 integrin. Biochem Cell Biol.

[20] Tsumagari T, Tanaka K (1984). Effects of fibrinogen degradation products on glomerular mesangial cells in culture. Kidney Int.

[21] Amdur RL, Feldman HI, Gupta J, Yang W, Kanetsky P, Shlipak M, Rahman M, Lash JP, Townsend RR, Ojo A, Roy-Chaudhury A, Go AS, Joffe M (2016). Inflammation and Progression of CKD: The CRIC Study. Clin J Am Soc Nephrol.

[22] Muslimovic A, Rasic S, Tulumovic D, Hasanspahic S, Rebic D (2015). Inflammatory Markers and Procoagulants in Chronic Renal Disease Stages 1-4. Med Arch.

[23] Celik IE, Kurtul A, Duran M, Yarlioglues M, Elcik D, Kilic A, Koseoglu C, Oksuz F, Murat SN (2016). Elevated serum fibrinogen levels and risk of contrast-induced acute kidney injury in patients undergoing a percutaneous coronary intervention for the treatment of acute coronary syndrome. Coron Artery Dis.

[24] Schei J, Stefansson VT, Mathisen UD, Eriksen BO, Solbu MD, Jenssen TG, Melsom T (2016). Residual associations of inflammatory markers with eGFR after accounting for measured GFR in a community-based cohort without CKD. Clin J Am Soc Nephrol.

[25] Kusano T, Takano H, Kang D, Nagahama K, Aoki M, Morita M, Kaneko T, Tsuruoka S, Shimizu A (2016). Endothelial cell injury in acute and chronic glomerular lesions in patients with IgA nephropathy. Hum Pathol.

[26] Trimarchi H, Barrat J, Cattran DC, Cook HT, Coppo R, Haas M, Liu ZH, Roberts IS, Yuzawa Y, Zhang H, Feehally J (2017). Oxford Classification of IgA nephropathy 2016: an update from the IgA Nephropathy Classification Working Group. Kidney Int.

[27] Haas M, Verhave JC, Liu ZH, Alpers CE, Barratt J, Becker JU, Cattran D, Cook HT, Coppo R, Feehally J, Pani A, Perkowska-Ptasinska A, Roberts IS (2017). A Multicenter Study of the Predictive Value of Crescents in IgA Nephropathy. J Am Soc Nephrol.

[28] Onozato ML, Tojo A, Kobayashi N, Goto A, Matsuoka H, Fujita T (2007). Dual blockade of aldosterone and angiotensin II additively suppresses TGF-beta and NADPH oxidase in the hypertensive kidney. Nephrol Dial Transplant.

[29] Ihm CG, Jeong KW, Lee SH, Lee TW, Park JK (2007). Effects of therapeutic agents on the inflammatory and fibrogenic factors in IgA nephropathy. Nephrology (Carlton).

[30] Romero CA, Orias M, Weir MR (2015). Novel RAAS agonists and antagonists: clinical applications and controversies. Nat Rev Endocrinol.

[31] Pozzi C (2016). Treatment of IgA nephropathy. J Nephrol.

[32] Snaedal S, Heimburger O, Qureshi AR, Danielsson A, Wikstrom B, Fellstrom B, Fehrman-Ekholm I, Carrero JJ, Alvestrand A, Stenvinkel P, Barany P (2009). Comorbidity and acute clinical events as determinants of C-reactive protein variation in hemodialysis patients: implications for patient survival. Am J Kidney Dis.

[33] Levey AS, Stevens LA, Schmid CH, Zhang YL, Castro AF, Feldman HI, Kusek JW, Eggers P, Van Lente F, Greene T, Coresh J (2009). CKD-EPI (Chronic Kidney Disease Epidemiology Collaboration). A new equation to estimate glomerular filtration rate. Ann Intern Med.

[34] Cattran DC, Coppo R, Cook HT, Feehally J, Roberts IS, Troyanov S, Alpers CE, Amore A, Barratt J, Berthoux F, Bonsib S, Bruijn JA, D’Agati V (2009). Working Group of the International IgA Nephropathy Network and the Renal Pathology Society. The Oxford classification of IgA nephropathy: rationale, clinicopathological correlations, and classification. Kidney Int.

[35] Ho D, Imai K, King G, Stuart EA (2011). MatchIt: Nonparametric Preprocessing for Parametric Causal Inference. https://doi.org/10.18637/jss.v042.i08.

[36] Team RC (2017). R: A Language and Environment for Statistical Computing.

[37] R Project (2017). rms: Regression Modeling Strategies.

